# Therapeutic potential of aromatic plant extracts in Alzheimer's disease: Comprehensive review of their underlying mechanisms

**DOI:** 10.1111/cns.14234

**Published:** 2023-04-30

**Authors:** Yue Ma, Yingming Li, Run Yin, Peixin Guo, Nai Lei, Gang Li, Lei Xiong, Yuhuan Xie

**Affiliations:** ^1^ Basic Medical School Yunnan University of Chinese Medicine Kunming China; ^2^ School of Chinese Materia Medica Yunnan University of Chinese Medicine Kunming China; ^3^ College of Ethnic Medicine Yunnan University of Chinese Medicine Kunming China; ^4^ Yunnan Provincial University Key Laboratory of Aromatic Chinese Herb Research Kunming China; ^5^ Yunnan Innovation Team of Application Research on Traditional Chinese Medicine Theory of Disease Prevention at Yunnan University of TCM Kunming China

**Keywords:** Alzheimer's disease, aromatic plant extracts, therapeutic potential, underlying mechanisms

## Abstract

**Aims:**

The aim of this review is to outline recent advancements in the application and mechanistic studies of aromatic plant extracts in Alzhermer`s disease (AD) to demonstrate their value in the management of this disease.

**Background:**

AD is a neurodegenerative disease with a complex pathogenesis characterized by severe cognitive impairment. Currently, there are very few drugs available for the treatment of AD, and treatments are primarily focused on symptom relief. Aromatherapy is a traditional complementary alternative therapy that focuses on the prevention and treatment of the disease through the inhalation or transdermal administration of aromatic plant extracts. Over the past few years, studies on the use of aromatic plant extracts for the treatment of AD have been increasing and have demonstrated a definitive therapeutic effect.

**Methods:**

We systematically summarized in vitro, in vivo, and clinical studies focusing on the potential use of aromatic plant extracts in the treatment of AD in PubMed, ScienceDirect, Google Scholar, and the Chinese National Knowledge Infrastructure from 2000 to 2022.

**Results:**

Our literature survey indicates that aromatic plant extracts exert anti‐AD effects by modulating pathological changes through anti‐amyloid, anti‐tau phosphorylation, anti‐cholinesterase, anti‐inflammation, and anti‐oxidative stress mechanisms (Figure 1).

**Conclusion:**

This review provides a future strategy for the research of novel anti‐AD drugs from aromatic plant extracts.

## Graphical abstract

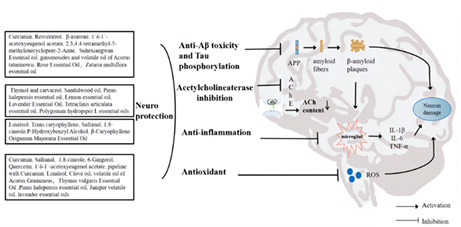

Highlights

- Alzheimer's disease (AD) is a complex multifactorial neurodegenerative disease for which only a few drugs are currently available as symptomatic treatment.
- Aromatic plant extracts are indeed effective in improving cognitive function, reducing restlessness, and improving sleep quality in AD patients.
- The pharmacological mechanisms of aromatic plant extracts to improve AD are suggested to be the reduction of Aβ and hyper‐phosphorylation of tau protein, anti‐cholinesterase, inhibition of the release of inflammatory cytokines, and reduction of damages of oxidative stress.
- Aromatic plant extracts are considered to have neuroprotective effects and have potential medicinal value for the treatment of AD.

## 1 INTRODUCTION

Alzheimer's disease (AD) is a neurodegenerative disease with a progressive and insidious onset characterized clinically by memory impairment, aphasia, dysfluency, impaired recognition and impaired visuospatial skills, performance dysfunction, character and behavior, along with other manifestations of general dementia. With global aging, AD incidence is increasing and is expected to impose heavy economic burdens on society. That the pathogenesis of AD remains unclear and also poses huge challenges in the study of this disease.

In terms of AD treatment, current drug research and development is based mainly on the neurotransmitter hypothesis and the amyloid cascade hypothesis. To date, the US. Food & Drug Administration has authorized six medicines for AD treatment: donepezil, memantine, rivastigmine, galantamine, tacrine, and aducanumab. The first‐line therapeutic agent in the primary phase of clinical treatment of AD is often donepezil, which is an acetylcholinesterase (AChE) inhibitor.^1^ During the first 3 months of dosing, patients showed improvement in cognitive symptoms. However, its effects gradually diminished as tolerance developed. N‐methyl‐d‐aspartate receptor agonists such as memantine are often applied to treat moderate to severe AD, and although they improve AD symptoms, they do not stop or delay disease progression.^2^

Since beta‐amyloid (Aβ) has a significant part in the pathological mechanism of AD and is an early trigger for the development of AD, enhancing Aβ clearance through Aβ‐targeted immunotherapy to reduce Aβ deposition to the brain and therefore, improve cognitive performance is a theoretically feasible strategy. Aducanumab, the world's first Aβ monoclonal antibody being used for the prevention of AD, can reduce Aβ deposition, delaying disease clinical progression in a drug‐dependent and time‐dependent way.^3^, ^4^ Over the past 5 years, non‐Aβ target drugs have also gained increasing attention, including targets for tau proteins, inflammation, synapses, and neuronal protection.^5^, ^6^ However, it is controversial whether the these therapeutic agents can slow down clinical cognitive deterioration in people with AD.

**FIGURE 1:**
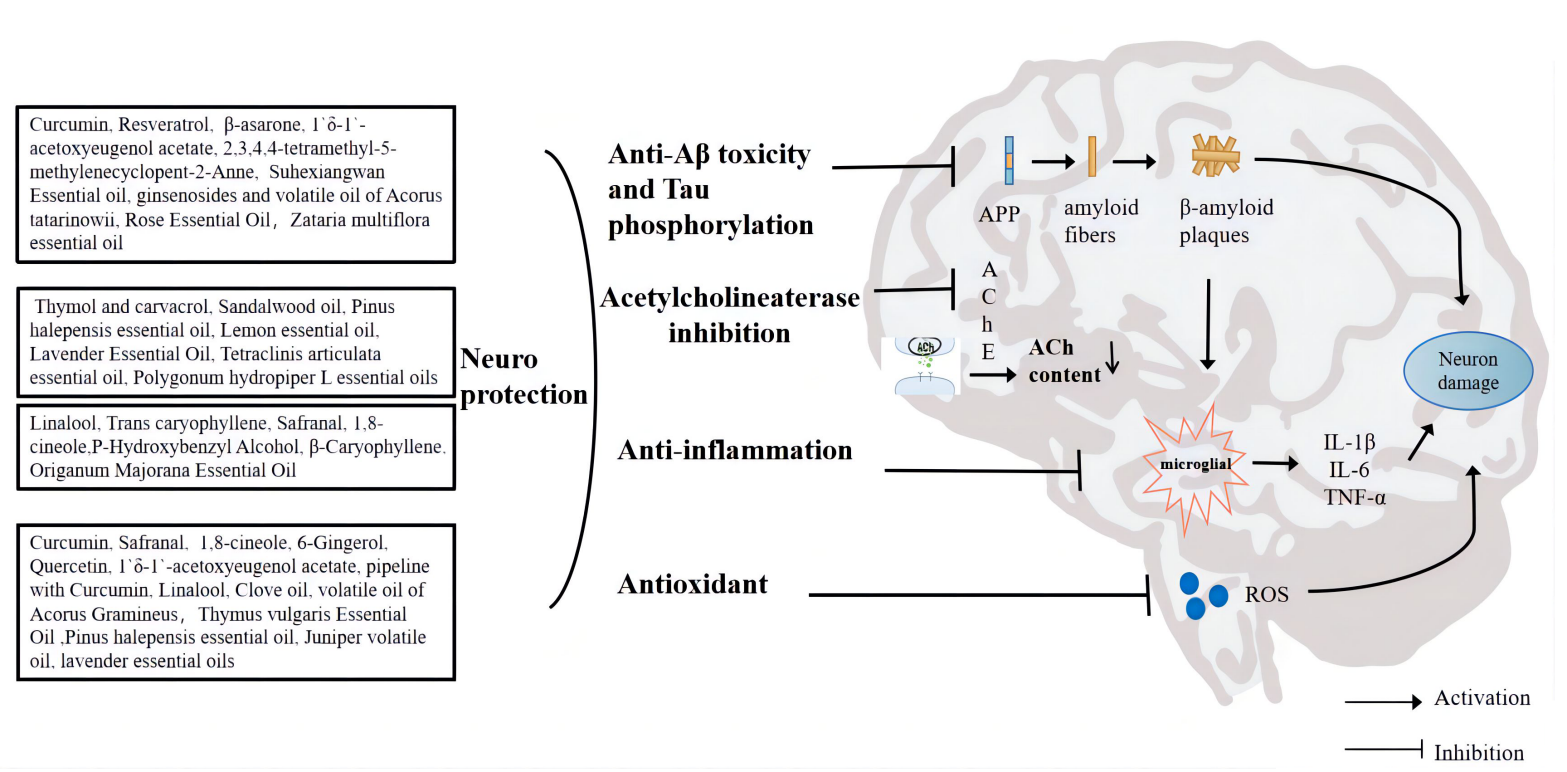
Potential mechanisms of aromatic plant extracts for the treatment of Alzheimer's disease. ↓‐reducing, Aβ, amyloid β protein; Ach, acetylcholine; AChE, acetylcholinesterase; APP, amyloid precursor protein; IL‐1β, interleukin‐1β; IL‐6, interleukin‐6; ROS, reactive oxygen species; TNF‐α, tumor necrosis factor α.

The pursuit of innovative and more potent drugs for the prevention of AD has been a hot topic in the field of drug development. The search for potential therapeutic drugs from traditional medical use experience has become a proven avenue for drug discovery. Essential oils are active components of aromatic plants and are abundant in leaves, seeds, flowers, bark, and roots.^7^ These complex, volatile, naturally occurring mixtures of compounds are secondary metabolites of the plant. The main chemical constituents of these aromatic products include monoterpene hydrocarbons, sesquiterpenes, oxygenated sesquiterpenes, oxygenated monoterpenes, and esters, etc. Essential oils are absorbed by the body in three main ways, through the respiratory system, the skin, and orally.^8^ Regardless of the administration route, aromatic compounds can easily enter the central nervous system (CNS) by inhalation or crossing the blood–brain barrier (BBB). It had already been indicated that aromatic components have a certain opening effect on the BBB in in vivo mice, and that the lipid position of aromatic constituents may be the material basis of their action on brain tissue.^9^ Therefore, essential oils have obvious advantages in the treatment of neurological disorders. Aromatherapy through the application of essential oils may constitute a possible treatment option for AD (Figure 1).

To evaluate the status of aromatic plant extract application during AD progression, we conducted PubMed, ScienceDirect, Google Scholar, and China national knowledge infrastructure searches of studies published between 2000 and 2022, with key phrases that included Alzheimer's disease, aromatic plant, aromatic compounds, essential oils, and neuroprotection. In this review, recent advances in clinical applications and mechanistic studies of aromatic plant extracts in AD will be outlined to illustrate the value of aromatic plant applications in this field.

## 2 BENEFICIAL EFFECTS OF AROMATIC PLANT EXTRACTS IN CLINICAL AD TREATMENT

Patients with AD experience age‐related cognitive decline, and commonly suffer from disturbed sleep patterns, anxiety, and agitation. These symptoms usually worsen with age and the severity of dementia. Current clinical research supports the applicability of aromatherapy in cognitive dysfunction and agitation.

In a study of 28 older adults, 17 of whom had AD, aromatherapy use consisted of morning rose and lemon essential oil (LEO) and evening lavender and orange for 28 days. After treatment, all patients showed significant enhancement in self‐reported cognitive presentation on the Japanese versions of both the Gott fries, Brane, Steen Scale, and the Type Dementia Assessment Scale.^10^ Lavender essential oil, popular due to its ease of application and minimal side effects, significantly reduces non‐aggressive physical behaviors in residential patients with dementia.^11^ A randomized clinical trial of 3 months of therapeutic massage using 2% lavender essential oil on selected acupuncture points in 60 AD patients was found to reduce the severity and distress of behavioral and psychological dementia symptoms.^12^ In another study assessing the effects of olfactory neurostimulation showed that cedar scent significantly improved Neuropsychiatric Inventory (NPI) and Zarit Caregiver Burden Interview (J‐ZBI) scores in patients with AD without olfactory dysfunction at 4 and 8 weeks.^13^ In a double‐blind placebo‐controlled research study in which AD patients with severe dementia, melissa essential oil therapy, or placebo was placed on the patients' faces and arms twice daily for 4 weeks. Results showed a 30% reduction in patients' Cohen‐Mansfield Agitation Scale (CMAI) scores and an overall improvement in agitation and quality of life parameters.^14^ Table 1 shows the list of beneficial effects of aromatic plant extracts in clinical AD treatment.

**TABLE 1:** Application of aromatic plant extracts in clinic of AD.

Composition	Number of patients	Treatment	Major Finding	Ref.
*Melissa officinalis* oil	72 AD patients	Faces and arms twice a day lasts for 4 weeks	↓CMAI score and quality of life reflected	14
*Melissa officinalis* oil	114 AD patients	Massaging the oil into the hands and upper arms lasts for 12 weeks	↑PAS ↓NPI	56
*Lavender augustofolia* essential oil	24 AD patients	Placed into fan diffuser for 3 months	Calm down and sleep more fully	57
Rosemary and lemon essential oils lavender and orange	28 AD patients	Placed into fan diffuser for 28 days	↑Personal orientation related to cognitive function on both the GBSS‐J and TDAS	10
A mixture of lemongrass essential oil and eucalyptus oil in a ratio of 2:1 suspended in jojoba oil	50 AD patients	Massage and inhalation for 4 weeks	↓CMAI ↓NPI	58
Cedar fragrance	36 AD patients were recognized as not having olfactory dysfunctions	Exposed to a disinfecting ethanol with added aroma extracts from ceder	↓NPI ↓J‐ZBI	13

Abbreviations: ↑‐increasing; ↓‐reducing; CMAI, Cohen‐Mansfifield Agitation In ventory; GBSS‐J, the Japanese version of the Gottfries, Brane, Steen scale; NPI, The Neuropsychiatric Inventory; PAS, Pittsburgh Agitation Scale; TDAS, type Dementia Assessment Scale.

The studies cited above provide evidence for clinical use of aromatic plant extracts. Currently, they are mainly applied in treatment by both massage and inhalation and are indeed effective in improving cognitive function, reducing restlessness, and improving sleep quality in AD patients. However, there are still deficiencies in the current clinical research. There is considerable heterogeneity in the available study designs, ranging from feasibility studies to controlled and non‐controlled studies, so that these studies differ in terms of the level of evidence and methodological quality. In addition, the outcome evaluation scales selected varied considerably across all studies. In some studies, doses of subcutaneous and inhaled applications were not quantified, and in others, participants were taking antipsychotics and comorbid medications that were not controlled. As a result, there is considerable variation in evaluating and assessing the therapeutic effects of essential oils. Thus, further high‐quality randomized controlled trials are needed to clarify the effect of aromatherapy in cognitive disorders. In addition, aromatherapy can be used as an alternative therapy, complementary alternative medicines need to be safe, routine laboratory tests need to be done before and after treatment, such as blood analysis and biochemical examinations.

## 3 BENEFICIAL EFFECTS OF AROMATIC PLANT EXTRACTS IN PRECLINICAL AD

### 3.1 Counteracting effects of aromatic plant extracts on Aβ toxicity and tau phosphorylation pathologies

It is well‐known that the characteristic pathology of AD is mainly the deposition of Aβ along with increased neuronal fiber tangles in the brain, so reducing Aβ deposition and inhibiting hyper‐phosphorylation of tau protein are essential aspects of anti‐Alzheimer's drug mechanism research. Animal experiments have shown that oral administration or inhalation of aromatic essential oils can effectively improve cognitive function in dementia animals by reducing Aβ deposition. A combination of lemon and rosemary oils at night and a combination of lavender and orange oils during the day for 2 months of inhalation could improve cognitive performance in senescence‐accelerated mouse prone 8 by reducing brain activity of Aβ and abnormally phosphorylated tau in the mouse brain.^15^ In another animal study, the authors reported that combined oral administration of total ginsenosides and essential oil of *Acorus tatarinowii* significantly increased learning memory and decreased Aβ1‐42 levels and reduced age spot formation in a D‐galactose‐ and aluminum chloride‐induced AD mouse model.^16^ SuHeXiang Wan (SHXW) is a conventional Chinese medicinal preparation usually used for the therapy of CNS disease. The formula consists mainly of aromatic herbs. Research has shown that pre‐inhalation of SHXW essential oil modified memory impairment caused by Aβ1‐42 and JNK, p38, and tau phosphorylation in the mouse hippocampus.^17^ In addition, SHXW essential oil has lipophilic fraction reacting with the lipid parts of cell membranes. It is more frequently used in topical applications rather than internal use. It suggests that SHXW essential oil may have future as an inhaled medicine for the prevention and treatment of AD.

In recent years, many constituents of aromatic plants have been tested for their potential to reduce symptoms of AD or for affecting the disease mechanism. Thymol(2‐isopropyl‐5‐methylphenol) and carvacrol(5‐isopropyl‐2‐methylphenol) are the main ingredients in the essential oils of numerous aromatic plants such as species of *Zataria*, *Origanum*, *Thymbra*, *Thymus*, *Satureja*, and *Coridothymus* of the family Lamiaceae and *Lippia* of Verbenaceae.^18^ Thymol or carvacrol may lightening cognitive impairment in rats received bilateral intrahippocampal injections of Aβ (25–35).^19^ Among Aβ‐related research items, the curcumin has received the most attention. Curcumin is a kind of phenolic compound obtained from the rhizome of some plants in the Zingiberaceae and Araneae families,^20^, ^21^ which is the major constituent of the Asian spice. Animal studies have shown curcumin could ameliorate cognitive impairment and reduced the hippocampal β‐amyloid accumulation in an i.c.v.‐streptozotocin Alzheimer's disease rat model.^22^ Lim confirmed that post‐treatment of curcumin on amyloid pathology in APPSw mice, plaque size was reduced by an average of 14%.^23^ Furthermore, Bisceglia demonstrated curcumin could inhibit the formation of toxic Aβ oligomers and limit the formation of insoluble fibrils.^24^ Moreover, curcumin has a variety of other biological activities related to the treatment of AD, such as anti‐inflammatory and antioxidant effects that will be described later. Based on multi‐functional properties of curcumin, therefore, could be a therapeutic option for the treatment of AD, provided limitations in its oral bioavailability can be overcome.

Accumulating evidence suggests that aromatic plant extracts can exert therapeutic effects by reducing plaque size, promoting Aβ clearance, and decreasing phosphorylated tau levels in both transgenic and chemically induced animal models of AD. However, further investigations are necessary to confirm its biological targets as well as potential toxicity before any recommendations can be made for its use as an anti‐dementia treatment.

### 3.2 Counteracting effect of aromatic plant extracts on pathologic AChE activity

Acetylcholine (ACh) has an irreplaceable role in the CNS. It was found that cholinergic neurons, located in the basal forebrain, are severely lost in the brains of AD patients, leading to deficits in memory and attention. ACh present in the synaptic cleft is hydrolyzed by AChE, and this pathological change is therefore thought to be associated with AChE activity. Therefore, inhibition of AChE activity has become a part of the therapeutic interventions for the prevention of AD. Competitive inhibitors of AChE are also the most widely used drugs for the prevention of AD.^25^

Several studies have been reported on the cholinesterase inhibitory potential of aromatic plant extracts. In vitro AChE activity was often measured following Ellman's assay. Essential oils (EOs) from *Salvia leriifolia* Benth. exhibited high BChE inhibitory.^26^ Volatile oil from *Marlierea racemosa* Vell. (Myrtaceae) demonstrated concentration‐dependent inhibition of AChE,^27^ EOs from the leaves and flowers of *Polygonum hydropiper* L.,^28^ sandalwood oil and its chief constituent α‐santalol were reported the AChE, BChE inhibitory efficacy.^29^

Apart from that, in vivo anti‐cholinesterase activities of aromatic plants and their extracts were also determined. The extract of *Rosmarinus officinalis* L. leaf led to improved long‐term memory in scopolamine‐induced rats, which can be partially explained by its inhibition of AChE activity in rat brain.^30^ In a rat model of acute Aβ_1‐42_ toxicity, Pinus halepensis essential oil reversed the performance of spontaneous alternation rate on Y‐maze test, increased spatial memory and restoration of false memories in the radial arm maze test, and reduced AChE activity in hippocampus.^31^ It was observed in APP/PS1 mice that 4 weeks of Lemon essential oil treatment could significantly decrease hippocampal AChE, and thus increased ACh levels.^32^

As mentioned above, the inhibition of AChE to increase the concentration of neurotransmitter is highly recommended. Some aromatic volatile oils and their major components showed significant cholinesterase inhibitory effects and may improve cognitive performance in AD model animals through this mechanism. Most studies have found that terpenoids in aromatic plant extracts are the main anticholinesterase active components, but their structural diversity complicates the study of structure–activity relationships.

### 3.3 Counteracting effects of aromatic plant extracts on inflammatory pathology

Increasing evidence suggests that damaged neurons and deposits of insoluble Aβ peptides and neurofibrillary tangles are pathological triggers for neuroinflammation in AD. When these substances bind to the pattern recognition receptors of microglia and astrocytes, they induce transcriptional activation of downstream inflammatory response genes, thereby releasing inflammatory cytokines, the overproduction of which eventually leads to chronic neuroinflammation, thus exacerbating the pathogenesis of AD.^33^ On the other hand, during peripheral inflammation, cytokines cross the BBB and cause brain microglia and astrocytes to react, further producing pro‐inflammatory mediators.^34^ The production of inflammatory cytokines and mediators by microglia and astrocytes directly promotes the occurrence and development of AD.^35^

Microglia and astrocytes play an important role of inflammatory responses in the CNS.^36^ Morphological changes in microglia and astrocytes can indicate neuroinflammatory response. Several aromatic plant extracts were found suppression of neuro‐inflammation in AD animal models. 6‐Gingerol, the major component of gingerols extracted from *Zingiber officinale*, was reported to attenuate LPS‐induced impairment of learning and memory in the Morris water maze in a dose‐dependent manner, and inhibition of LPS‐induced increases in astrocytic marker GFAP and TNF‐α levels in the rat brain was suggested as the mechanism.^37^ Linalool is a natural monoterpene and the main component of the EOs of plants such as *Lavandula angustifolia* Mill., *Melissa officinalis* L., *Rosmarinus officinalis* L., and *Cymbopogon citratus* DC. Oral administration of the monoterpene linalool for 3 months AD triple transgenic model mice (3xTg‐AD) exhibited a dramatically diminished in astrogliosis and microgliosis in the hippocampus and amygdala.^38^ Oral administration of curcumin could lower level of GFAP and inhibit microgliosis in cortical and hippocampal layers in the APPSw mice.^23^

The inhibition or downregulated expression of key pro‐inflammatory mediators and/or the upregulation of anti‐inflammatory cytokines are beneficial for preventing chronic inflammation and further cell death. Studies have shown that linalool,^38^ safranal,^39^ 6‐Gingerol,^37^ and curcumin^23^ could inhibit pro‐inflammatory cytokines such as tumor necrosis factor‐alpha (TNF‐α), interleukin (IL)‐1β, IL‐6, and IL‐8 to reduce neuroinflammation in AD animal models. Oral administration of the monoterpene linalool could reverse spatial memory impairment in old 3xTg‐AD mice, downregulate of proinflammatory cytokines and p38 mitogen‐activated protein kinase (MAPK) in the hippocampus, suggesting that linalool exerts its effect on restoring cognitive function probably by counteracting the inflammatory response.^38^ Safranal is an active ingredient of Crocus sativus volatile oil, which improved cognitive abilities in intrahippocampal a Aβ1‐40 induced a rat model of AD, and reduced content of IL‐6, TNF‐α, and IL‐1β in hippocampal tissue.^39^

Moreover, the transcription factors that regulate chronic inflammation in AD are nuclear factor kappa‐activated light chain B (NF‐κB), which positively regulate several genes that encode proinflammatory cytokines and can also be used as inflammatory markers.^40^ It has been shown that Safranal could prevent learning and memory decline by inhibiting or downregulating NF‐κB translocation.^39^ Trans‐caryophyllene, a main constituent of volatile oils of *Syzygium aromaticum*, *Rosmarinus offificinalis*, and *Cannabis sativa*, was shown to reduce the phosphorylation and degradation of NF‐κB inhibitor α in BV‐2 microglia treated with Aβ_1‐42_. Aβ is known to promote neurodegeneration through TLRs to activate microglia and astrocytes and release neuroinflammatory mediators. It is reported that trans‐caryophyllene's remarkably attenuated Aβ_1–42_ activated overexpression of toll‐like receptor 4 (TLR4) in BV‐2 microglial cells.^41^

COX‐2 is also involved in neurodegenerative diseases.^42^ The non‐steroidal anti‐inflammatory (NSAIDs) may reduce microglial activation.^43^ Hu Y et al.reported trans‐caryophyllene decreased the Aβ_1–42_ activated overexpression of COX‐2 and prostaglandin E_2_ (PGE_2_) generation.^41^ In another study, linalool‐treated 3xTg‐AD mice exhibited a significant reduction in COX‐2 in hippocampi and amygdalae from 3xTg‐AD mice.^38^

The involvement of the inflammatory response in the pathological process of AD has been repeatedly demonstrated. However, clinical trials with anti‐inflammatory drugs have not been successful.^44^ This suggests that the complex neuroinflammatory pathological process cannot be repaired by intervening at a particular target. Aromatic plants are used in traditional aromatherapy to treat a variety of health conditions, including pain and inflammation. Essential oils obtained from aromatic plants are widely used in complementary therapies for the treatment of complex inflammatory diseases such as arthritis and obesity. Many aromatic plant extracts, especially essential oils, have been proposed in preclinical studies, with results on the major mechanisms involved in the pathology of inflammation. There is still insufficient evidence to draw conclusions on anti‐inflammatory properties of aromatic plants without promising outcomes from clinical trials. Based on the results of animal studies mentioned above, we speculate that the anti‐inflammatory effects of aromatic plants are likely to have multi‐target effects, which are more advantageous than other single‐target drugs such as NSAIDs for the treatment of complex inflammatory diseases. In addition, the limitation of these studies was how long its effects persist. Such analyses and studies are strongly warranted to be included and/or conducted in future.

### 3.4 Counteracting effects of aromatic plant extracts on oxidant pathology

The CNS is highly vulnerable to oxidative stress, due to its high metabolic activity, elevated oxygen requirement, and the presence of high levels of redox‐active metals and oxidizable lipids. Oxidative stress is essential in the pathogenesis of AD. Aβ and cell damage induce the chronic production of reactive oxygen species (ROS) in the brains of AD patients. Excessive ROS and reactive nitrogen species (RNS) can lead to neuronal damage and are thought to be a mechanism for the pathogenesis and progression of AD.^45^ In contrast to oxidant mechanisms, the organism has endogenous defense antioxidant systems, including for example superoxide dismutase (SOD), glutathione peroxidase (GSH‐Px), and catalase (CAT). When ROS production is greater than cellular antioxidant capacity, oxidative stress can damage DNA, proteins, and lipids.^46^

Researchers have studied various aromatic plant extracts for their potent antioxident and free radical scavenging properties. The enhancement of endogenous antioxidant systems is often mediated via the activation of the nuclear factor‐like 2 (Nrf2) signaling pathway which regulates the gene expression of antioxidant enzymes. An in vitro study showed that SHXW essential oil could inhibit Aβ_1‐42_ induced apoptosis and ROS production by upregulating heme oxygenase 1 (HO‐1) and Nrf2 expression in SH‐SY5Y cells.^17^

Several compounds like safranal, linalool, and SHXW essential oil have been found to decrease ROS levels induced by Aβ in rats or mouse. Safranal attenuated MDA levels in the hippocampus and increased SOD activity.^39^ Linalool administration attenuated the neurotoxicity of Aβ by reducing nitric oxide (NO) levels.^47^

Furthermore, the restoration of GSH‐Px, SOD, and CAT by curcumin,^48^ clove oil,^49^ Acorus Gramineus essential oil,^50^ lavender essential oils,^16^ and Juniper volatile oil^51^ signifies an increase in endogenous antioxidant defenses, which in turn prevents protein oxidation.

Curcumin pre‐treatment significantly has been found to modify the alterations in RNS levels in quinolinic acid‐induced behavioral and neurological changes in rats.^48^ Clove oil is a mind stimulant used in the treatment of anxiety, insomnia, and depression,^49^ which could reverse memory impairment in rats with i.c.v. colchicine‐induced memory disfunction by reducing lipid peroxidation levels, nitrite level, and restored glutathione levels.^52^ Stimulation of olfaction with the essential oil of Acorus Gramineus on i.c.v. Aβ1‐40 induced rat model significantly increased SOD and GSH‐Px activities and decrease CNS malondialdehyde (MDA) content.^50^

Co‐administered total ginsenosides and volatile oil of *Acorus tatarinowii*, showed a reduction in cortical and hippocampal MDA and a remarkable increase in SOD activity in a mouse model of D‐galactose and aluminum chloride.^16^ Lavender essential oils extracted from *Lavandula angustifolia* ssp. *angustifolia Mill*. and *Lavandula hybrida Rev*. have been found to increase the antioxidant enzymes activity including SOD, GPX, and CAT, while decrease the total GSH and MDA content in rat temporal lobe homogenates , indicating their antioxidant potential.^53^

Juniper volatile oil is extracted from *Juniperus communis* L., of the Cupressaceae family. Cioanca O et al determined the influence of the juniper volatile oil on AD rat which received a single i.c.v. injection of Aβ_1–42_ and then were exposed to juniper volatile oil. Similarly, the antioxidant potential of juniper volatile oil in the hippocampus was also comfirmed by using SOD, GSH‐Px, and CAT activities, the total content of the reduced glutathione and protein carbonyl levels.^51^ Of concern is that the safety of juniper volatile oil  need to be properly addressed. It has been reported a little toxicity of the juniper volatile oil extracted from multiple juniper species in animals.

The antioxidant effect of aromatic plant extracts due to direct scavenging of free radicals and increasing antioxidant capacity. This evidence is particularly strong in the aromatic components of polyphenols and terpenes. Polyphenols and essential oil terpenes protect cellular components from oxidative damage, thereby decrease the danger of diseases associated with oxidative stress. Table 2 shows the list of beneficial effects of aromatic plant extracts in preclinical AD.

**TABLE 2:** Application of aromatic plant extracts in the preclinic.

Composition	Models	Treatment	Behavior Test	Major Finding	Ref.
*Acorus tatarinowii* Schott Essential Oil	Aβ‐induced paralysis in transgenic CL4176 worms	Cultured for 36 h at 16°C containing 10 mg/mL then to 23°C, 32 h		↓Aβ ↓Polk Accumulation ↓ROS ↑autophagy‐related genes (log‐1, log‐2, bec‐1, vps34, and unc‐51), ↓P62 protein	^59^
Rose essential oil	Wild type strain N2, and the transgenic nematode strains	5 mg/mL cultured at 15°C for 84 h, then transferred to 25°C for 36 h		↓Aβ_1–42_ ↑SKN‐1 nuclear distribution ↑GST‐4	^60^
*Thymus vulgaris* essential oil	So‐induced zebrafish model 3–4‐month‐old, and 3–4 cm‐lon	25, 150, and 300 μL/L was administered by immersion to zebrafish once daily for 13 days	Y‐maze NOR NTT	↓AChE ↑SOD ↑GPX ↑GSH ↓MDA	^61^
Lavender essential oil	C57BL/6J mice (12 weeks) i.p. D‐gal and AlCl_3_	i.p. 50, 100 mg/kg/day for 8 weeks	OFT MWM PAT	↑SOD, ↑GPX ↓MDA ↓AChE ↑HO‐1 ↑Nrf2 ↑CaMKII ↑p‐CaMKII ↑BDNF ↑TrkB	^62^
Lemon essential oil	Male C57BL/6 and APP/PS1 mice (8 months)	1 mL/cage inhalation in the IVC for 1 h over 30 days	MWM NOR	↓AChE ↓APP ↑BDNF ↑TrkB ↑p‐AKT ↑p‐ERK ↑PSD95 ↑synapsin‐1	^32^
*Zataria multiflora Boiss*. essential oil	Male albino Wistar rats Aβ_25–35_ was injected bilaterally in the CA1 region of rats' hippocampus	i.p. 50, 100, or 200 μL/kg for 5 days	MWM	↓Mean escape latencies ↓Mean heads angles	^63^
*Zataria multiflora* essential oil	Adult male Sprague Dawley rats i.c.v. Aβ _1–42_	Orally 100 μL/kg/day for 20 days	MWM	↓Tau ↓TNF‐α	^64^
*Origanum majorana* essential oil	Adult male Wistar rats i.c.v. 4 μL of Aβ_1‐42_ solutions	Electronic vaporizer 200 μL for 15 min each day, for 21 days	Y‐maze RAM	↑BDNF ↓MDA ↓IL‐1β ↓Protein carbonyls	^65^
*Polygonum hydropiper* L. essential oils				↓AchE ↓ABTS ↓BChE ↓H_2_O_2_	^66^
*Tetraclinis articulata* essential oil	Male Wistar rats i.c.v. Aβ_1‐42_	Inhalation once daily for 15 min period at doses of 1% and 3% for 21 days	Y‐maze RAM	↓AChE ↑SOD ↑CAT ↑GSH‐Px ↑GSH ↓MDA ↓protein carbonyl	^67^
*Pinus halepensis* essential oil	Wistar rats‐males i.c.v. Aβ_1‐42_	Oil vapors, 15 min each day, for 21 days	Y‐maze RAM	↑SOD ↑CAT ↑GSH‐Px ↑GSH ↓protein carbonyl ↓MDA	^31^
Co‐administration of total ginsenosides and volatile oil of *Acorus tatarinowii*	Kunming (KM) mice were given D‐galactose, i.p, and aluminum chloride, i.g.,once daily for 40 days	i.g., Once daily for 40 days	MWM avoiding darkness experiments	↓AChE ↑ChAT ↓MDA ↓Aβ_1‐42_ ↑BCl‐2	^16^
Lavender oil	Male Wistar rats (i.p.) scopolamine daily, for 7 continuous days	Oil vapors for 60 min period, daily, for 7 days		↑CAT ↓GSH ↑GSH‐Px ↓MDA ↑SOD ↑SOD	^53^
SuHeXiang Wan (SHXW) essential oil	Male 8‐week‐old ICR mice i.c.v. Aβ_1‐42_	Oil (2 g) was inhaled twice a day for 3 h in the morning and afternoon for 14 days	Y‐maze RAM	↓Bax ↑Bcl‐2 ↓ ROS ↑HO‐1 ↑Nrf2 ↓p‐p38 ↓p‐JNK ↓p‐Tau	^17^
Lemon, rosemary, orange, and lavender essential oil	Male SAMP8/TaSlc mice (3 months of age)	Inhalation at nighttime and daytime for 2 months	Y‐maze	↓Aβ ↑BDNF ↓p‐Tau	^15^
Trans‐caryophyllene	Microglial cells treated with Aβ_1–42_	Pretreatment of BV2 microglia with 10, 25, and 50 μM for 24 h		↓NO ↓PGE2 ↓iNOS ↓COX‐2 ↓NF‐κB ↓LDH ↓TNF‐α ↓IL‐6 ↓ IL‐1β	^41^
1,8‐Cineole	PC12 cells treated with Aβ_25–35_ (0.01–10 μM)	Pretreated at 0–10 μM for 24 h		↓ROS ↓ NO ↓TNF‐α ↓IL‐1β ↓IL‐6 ↓NOS‐2 ↓COX‐2 ↓NF‐κB.	^68^
β‐Asarone	PC12 cell treated with 7.7 μM Aβ_1‐42_ for 6, 12, 24, 48 h	Pretreatment 24, 36, 72 μM for 24 h		↓APP ↓ PS1 ↓Aβ ↓BACE1 ↓p62 ↓Beclin‐1 ↑BECN1 ↑LC3 ↑SYN1	^69^
Gingerol	Streptozotocin‐induced sporadic AD Adult male Swiss albino mice	i.p.10 mg/kg and 20 mg/kg, for 7 days	MWM Y‐maze with intra‐maze cues	↓Aβ42 ↑α‐secrets activity ↓β‐ secrets activity ↓APH1a activity ↓COX‐2	^70^
6‐Gingerol	SH‐SY5Y cells treated Αβ _25–35_	Retreatment of 10 μM for 24 h		↑GCL ↑HO‐1 ↑Mitochondrial transmembrane Potential ↑Nrf2 ↑ Bcl‐2 ↓Caspase‐3	^71^
6‐Gingerol	C6 rat astroglioma cells, Adult male Sprague–Dawley rats LPS‐induced AD	i.p. once daily for 2 weeks	MWM	↓ROS ↓NO ↓GFAP ↓TNF‐α ↓iNOS ↓IL‐6	^37^
6‐Gingerol	PC12 cells treated with Aβ_1–42_ (5, 10, and 20 μM) for 24, 48, and 72 h	Pretreatment 40, 80, 120, 200, and 300 μM for 4 h		↓ROS ↓MDA ↓NO ↓LDH ↑SOD ↑p—Akt ↑p‐GSK‐3 β	^72^
Quercetin	Aβ_25‐35_ treated rat hippocampal neuron cells at 5 μM	0.1, 1, 10, 50, and 100 μM cultured in neurons for 72 h		↓GSH ↑NEP	^73^
Resveratrol	HEK293 cells stably transfected with human APP695	Treated with 10 or 20 μM resveratrol 48 and 72 h		↑ECE‐1 ↑ECE −2 ↑IDE	^74^
Linalool	21–24 months old 3xTg‐AD (Ps1M146V, APPswe, Taup301L) mice	i.p. 25 mg/kg every 48 h for 3 months	EPM EPDM	↓Aβ ↓MAPK pro‐inflammatory markers p38, ↓NOS2 ↓COX2 ↓IL‐1β	^38^
Curcumin	APPswe/PS1dE9 mice	Orally 100, 200, 400 mg/kg/day for 3 months	MWM	↓Aβ40 ↓Aβ42 ↓presenilin‐2, ↓β amyloid‐degrading enzymes	^75^
Curcumin	Aβ_25‐35_ cultured rat primary prefrontal cortical neuron cells	Pretreatment 1, 10, 20, and 40 μM for 24 h		↑Bcl2 ↓caspase‐3	^76^
P‐Hydroxybenzyl alcohol	Aβ_42_ induced Two‐month‐old ICR mice	i.g. 5, 15 mg/kg once a day for 18 days	NOR MWM	↑BDNF ↑GDNF ↓TNF‐α ↓IL‐1β ↑Nrf2	^77^
β‐Caryophyllene	7 months male APP/PS1 mice	Orally 16, 48, or 144 mg/kg for 10 weeks	MWM	↓COX‐2 ↓TNF‐α ↓IL‐1β ↓Aβ ↓Iba‐1	^78^
1‘δ‐1’‐Acetoxyeugenol acetate	Aβ _25–35_‐induced neurodegeneration in Male albino mice (Swiss) aged 6 weeks	Orally, 12.5 mg/kg, 25 mg/kg, 50 mg/kg for 28 days	OFT MWM step‐ Down inhibitory tests	↓AChE ↓TNF‐α, ↑Corticosterone ↑serotonin ↑DA ↓SOD ↓CAT ↑GSH‐Px ↑vitamin C	^79^
Gallic acid	APP/PS1 transgenic AD mouse model (12 months)	Orally 20 mg/kg once daily for 6 months	Y‐maze NOR RAWM	↓Aβ ↓GFAP ↑α‐ secretase activity (ADAM10) ↓β‐secretase activity (BACE1) ↓Iba1	^80^
β‐Asarone	APP/PS1 mouse (3 months)	Orally 10 mg/kg for 30 days	MWM PAT step‐down tests	↓Aβ_42_ ↓APP ↓Beclin‐1 ↑P‐Akt ↑P‐Mtor ↓AChE ↑PI3k	^81^
Tenuigenin and β‐asarone curcumin	APP/PS1 mice (3 month)	Orally 74 mg·kg for 2 weeks	NOR MWM	↑p‐Akt ↑p‐GSK‐3β	82, 83
	Male Sprague–Dawley rats i.c.v. Aβ_1‐42_	i.p. 50, 100, and 200 mg/kg for 7 days	MWM Y‐maze OFT	↑BDNF ↑P‐ERK	82, 83
Linalool	Aβ_1–42_ induced AD rat	i.p. once a day for 21 days		↓ROS ↓4‐HNE	^47^
2,3,4,4‐Tetramethyl‐5‐methylenecyclopent‐2‐Anne	CHO‐APPwt cells, SH‐SY5Y‐APPwt cells and 3xTg‐AD mouse aged 4–5 months	Administered by the intranasal route, once a day, for 4 days	MWM Rota‐rod accelerating test	↓Aβ_40_ ↓BACE‐1 ↓Cathepsin D	^84^
Thymol and carvacrol	Male albino Wistar rats and male NMRI mice injections of Aβ (25–35) or i.p. scopolamine	i.p. 0.5, 1, or 2 mg/kg for 5 days	MWM	↓Quadrant entries ↑Target quadrant entries	^19^

Abbreviations: ↑‐increasing; 5‐HT, 5‐hydroxytriptamine; ↓‐reducing; AChE, acetylcholinesterase; AD, Alzheimer diseases; AEL, average escape latency; Aβ, Amyloid β protein; Aβ1–42, the 42 amino acid form of amyloid β; BACE1, beta‐secretase 1; Bax, bcl2‐Associated X; Bcl‐2, B‐cell lymphoma‐2; BDNF, brain‐derived neurotrophic factor; CAT, catalase; COX‐2, e‐cyclooxygenas2; DA, dopamine; EPM, Elevated plus maze; GFAP, the astrocytic inflammatory marker; GSH, glutathione; GSH‐Px, glutathione peroxidase; HO‐1, nuclear heme oxygenase‐1; i.g., intragastric; i.p., inpraperitoneal; IL‐1β, interleukin‐1β; iNOS, Inducible nitric oxide synthase; IVC, individual‐ventilated cages; LDH, lactate dehydrogenase; MDA, Malondialdehyde; MWM, morris water maze; NE, norepinephrine; NF‐κB, nuclear factor; NO, nitric oxide; NTT, novel tank diving test; OFT, open field test; PAT, passive Avoidance Task; RAM, radial arm‐maze tests; ROS, reactive oxygen species; Sco, scopolamine; SOD, superoxide dismutase; TNFα, tumor necrosis factor‐α.

## 4 CONCLUSIONS AND FUTURE DIRECTIONS

Aromatherapy is a traditional complementary alternative therapy that focuses on the prevention and treatment of disease through the inhalation or transdermal administration of aromatic plant extracts. Clinical studies have also shown that aromatic plant extracts can improve cognitive function, reduce agitation, and improve sleep quality in AD patients. These volatile substance‐based components are thought to enter the bloodstream mainly through the respiratory mucosa, then through the BBB to the brain, and even diffuse directly to the olfactory nerve to reach the brain and exerting neuroprotective effects through multiple targets and pathways.

In this review, the pharmacological mechanisms of aromatic plant extracts to improve AD are suggested to be the reduction of Aβ and hyper‐phosphorylation of tau protein, anti‐cholinesterase, suppresses the release of multiple inflammatory cytokines caused by the deposition of Aβ and clears of free radicals, and reduction damages of oxidative stress. Among them, inflammation can trigger the overproduction of Aβ and tau proteins, which in turn induces an inflammatory response, leading to a vicious cycle of neuroinflammation and pathology. This review found that aromatic plant extracts had significant effects on improving inflammation, therefore, aromatic plant extracts are considered to have neuroprotective effects and have considerable medicinal value for the therapy of AD. However, the above conclusions were mainly obtained from animal or cellular experiments, and further clinical trials are needed to verify the above mechanisms. In addition, special attention must be paid to the kinetic characteristics, routes of administration, and doses of aromatic plants and their extracts in vivo and in vitro studies.

Furthermore, the ability of the hippocampus to retrieve memories is impaired in AD patients, many older people with AD are hypersensitive to sounds, smells, or temperature in their environment, and unfamiliar strong odors may overstimulate AD patients. However, earlier life memories rely less on the hippocampus for recollection, so it is highly likely that some older people with AD would recognize and may be comforted by their familiar fragrance, then have more effective and better tolerated. In addition, a correlation between olfactory function and brain regions involved in cognition and memory has been reported. Pathologically, amyloid beta protein accumulates in the olfactory nerve at the onset of Alzheimer's disease, and thus olfactory impairment is often observed before cognitive decline occurs in AD patients.^45^ There is progressive systemic damage to the olfactory nerve, causing olfactory impairment, which further leads to taste disturbance and loss of appetite, affecting the patient's quality of life. Appropriate olfactory stimuli can be transmitted through the olfactory nerve and be transmitted to the limbic system, including the hypothalamus.^54^ Possibly, the essential oils contain chemical constituents with bioactive properties that facilitate the recovery of the olfactory sense by suppressing inflammation and enhancing regeneration.^55^ Since the limbic system is closely related to cognition, olfactory stimulation may improve cognitive impairment.

## AUTHOR CONTRIBUTIONS

MY and YHX contributed to the conception, design, and preparation of the manuscript. NL, RY, and YL outlined the initial literature review and interpretation of published data. LX, PG, and GL made substantial contributions in drafting the manuscript and revising it critically for valuable intellectual content. All authors have read and approved the final version of the manuscript.

## FUNDING INFORMATION

This study was financially supported by the National Natural Science Foundation of China (NSFC) [No.82060823,82074421]. Natural Science Foundation of Yunnan Province [No.2017FA045 ]. The Yunnan Key Laboratory of Formulated Granules [No.202105AG070014].

## CONFLICT OF INTEREST STATEMENT

The authors declare that the research was conducted in the absence of any commercial or financial relationships that could be construed as a potential conflict of interest.

## Data Availability

Data openly available in a public repository that issues datasets with DOIs.
